# Can machine learning support infection control measures by predicting carbapenemase-producing Enterobacterales colonization at admission?

**DOI:** 10.1017/ice.2026.10440

**Published:** 2026-06

**Authors:** Shuk-Ching Wong, Edwin Kwan-Yeung Chiu, Jonathan Daniel Ip, Simon Yung-Chun So, Kelvin Hei-Yeung Chiu, Edmond Siu-Keung Ma, Kwok-Yung Yuen, Vincent Chi-Chung Cheng

**Affiliations:** 1 Infection Control Team, Queen Mary Hospital, Hong Kong West Cluster, Hong Kong Special Administrative Region, China; 2 School of Nursing, Li Ka Shing Faculty of Medicine, The University of Hong Kong, Pokfulam, Hong Kong Special Administrative Region, China; 3 Department of Microbiology, School of Clinical Medicine, Li Ka Shing Faculty of Medicine, Pokfulam, The University of Hong Kong, Hong Kong Special Administrative Region, China; 4 Department of Microbiology, Queen Mary Hospital, Hong Kong Special Administrative Region, China; 5 Centre for Health Protection, Department of Health, Hong Kong Special Administrative Region, China

## Abstract

**Background:**

Early identification of patients with carbapenemase-producing Enterobacterales (CPE) colonization is crucial for infection control; however, microbiological testing may delay detection and be costly. Machine learning may enhance predictive analytics for timely identification of at-risk patients.

**Methods:**

Four machine learning models: Decision Tree, Random Forest, Gradient Boosting, and XGBoost, were used to predict CPE colonization within 48 hours of admission using microbiological and demographic data. Model performance was assessed through sensitivity, specificity, and area under the receiver operating characteristic curve (AUROC). Uniform Manifold Approximation and Projection (UMAP) evaluated topological separability of CPE-positive cases and CPE-negative controls.

**Results:**

From January 1, 2015 to December 31, 2024, 453,372 fecal specimens were submitted for CPE screening, with 194,917 (43.0%) collected within 48 hours of admission, comprising 3,328 CPE-positive cases (1.7%) and 191,589 CPE-negative controls. The Gradient Boosting classifier showed the best performance, achieving an AUROC of 0.598, sensitivity of 54.4%, and specificity of 59.1%. Demographic factors (age ≥ 75 and male sex), history of hospitalization, and known CPE colonization in the past year, and admission specialty (general medicine and general surgery) were consistently included in all models as top predictors. UMAP revealed significant overlap between CPE-positive and CPE-negative patients, indicating challenges in differentiating the risk profiles.

**Conclusions:**

This study highlights the complexities of using machine learning to predict CPE colonization within 48 hours of admission. The low AUROC values suggest that the models may not effectively predict CPE colonization at the patient level, potentially due to inherent rarity of events and overlapping risk profiles.

## Introduction

Carbapenemase-producing Enterobacterales (CPE) have emerged as significant threats to global public health, with increasing prevalence in both healthcare and community settings.^1–4^ These antimicrobial-resistant organisms challenge infection control preventionists in hospitals, where plasmid-mediated carbapenemase can transfer between bacterial strains, facilitating resistance spread and patient-to-patient transmission, leading to hospital outbreaks.^5,6^ Patients with invasive CPE infections are associated with increased morbidity and mortality.^7,8^ Early identification of CPE colonization in hospitalized patients is crucial for implementing effective infection control measures to prevent nosocomial transmission.^9^

Traditionally, microbiological cultures have been the standard method for detecting CPE colonization;^9^ however, the time taken for laboratory diagnostics can delay infection control interventions. Expedited recognition of at-risk patients could greatly enhance the efficacy of infection control strategies by promoting timely isolation and management.

Recent advancements in machine learning offer promising alternatives for improving predictive analytics in healthcare settings.^10^ By leveraging extensive historical data from microbiology laboratories, machine learning algorithms may identify patterns and risk factors associated with CPE colonization, complementing standard surveillance by prioritizing high-risk patients for testing, thereby conserving resources and improving infection control efficiency.

In this study, we explore the potential of machine learning to support infection control measures by predicting CPE colonization within 48 hours of admission. By analyzing a decade of microbiological data from a university-affiliated teaching hospital, we aim to assess the effectiveness of machine learning models in anticipating CPE colonization. Our findings may provide insights into the feasibility of integrating machine learning in clinical workflows and enhancing proactive infection prevention efforts.

## Material and methods

### Setting

This study was conducted at Queen Mary Hospital, a 1,700-bed university-affiliated tertiary care facility in Hong Kong, providing comprehensive and specialized services, including unique centers for liver, heart-lung, and blood and marrow transplantation. Proactive infection control measures have been implemented for emerging pathogens,^11^ and epidemiologically important multidrug-resistant organisms (MDROs).^12–14^ To control CPE, admission screening strategies have evolved from targeting high-risk patients, defined as those with a known history of CPE colonization in the past year, admissions from local hospitals in the past 3 months, or hospitalization outside Hong Kong in the past year, to universal screening through the collection of fecal specimens (rectal swabs or stool samples) sent immediately to the microbiology laboratory for culture.^15^ The laboratory diagnosis process for CPE has been previously described.^15^ Laboratory reports are printed simultaneously in wards and the infection control office, allowing infection control nurses (ICNs) to coordinate with clinical staff for timely infection control measures, including enforcing hand hygiene, appropriate patient placement through cohort nursing, and implementing contact precautions.

### Data source and data extraction of patients with CPE colonization

All laboratory requests for detecting CPE, regardless of the presence or absence of CPE, were retrieved from the laboratory information system during the study period. Fecal specimens testing positive for carbapenem-resistant Enterobacterales (CRE), confirmed through either in-house molecular tests, the Xpert® Carba-R assay (Cepheid, Sunnyvale, CA, USA), or the lateral flow assays, NG-Test CARBA 5 (NG Biotech, Guipry, France) for carbapenemase-producing enzymes, were classified as CPE-positive cases. In contrast, fecal specimens testing negative for CRE were defined as CPE-negative controls. Only fecal specimens collected within 48 hours of admission were included to specifically target colonization status present at admission rather than nosocomial acquisition. If multiple specimens were submitted within this time frame, only the first specimen from each patient was analyzed.

The episode-based records of all cases and controls were retrieved from the Clinical Data Analysis and Reporting System (CDARS) as previously described.^16,17^ The input features included all available structured data in the CDARS system at the time of admission: age, sex, known history of CPE colonization in the past year, admission specialty (emergency medicine, general medicine, respiratory medicine, cardiology, nephrology, hematology, pediatrics, obstetrics and gynecology, general surgery, orthopedics and traumatology, cardiothoracic surgery, neurosurgery, oncology, liver transplantation, blood and marrow transplantation, and the intensive care unit), admission from residential care homes for the elderly (RCHE), history of hospitalization, endoscopy (upper or lower gastrointestinal tract, hepatobiliary system), proton pump inhibitors (PPIs) use, antimicrobial use (fluoroquinolones, cephalosporins, and carbapenems) in the past year, and chronic comorbidities (hypertension, diabetes mellitus, renal failure, and malignant tumor). These variables were selected as they represent accessible epidemiological risk factors in the electronic health record during admission.

### Prediction of CPE colonization within 48 hours of admission by machine learning

Four machine learning models were selected to predict CPE colonization in patients within 48 hours of admission: Decision Tree, Random Forest, Gradient Boosting, and XGBoost. Decision Tree is a fundamental model that splits data into branches based on feature values to reach a conclusion. Random Forest improves upon this by creating an ensemble of many decision trees to reduce overfitting. Gradient Boosting and XGBoost are advanced ensemble techniques that build models sequentially, where each new model corrects the errors of the previous ones, typically offering higher predictive accuracy. Patient demographic and clinical data were input into these models, with 80% of the data set used for training and 20% for testing via stratified random sampling to maintain the ratio of CPE-positive cases to CPE-negative controls. To address the issue of class imbalance within the data set, the Synthetic Minority Over-sampling Technique (SMOTE) was applied to the training data. The predictive results, whether using all demographic data collectively or each parameter individually, were validated against microbiology laboratory confirmations.

### Machine learning model development and implementation

Machine learning performance was evaluated using a comprehensive suite of metrics, including accuracy, sensitivity, specificity, positive predictive value (PPV), and negative predictive value (NPV). Discrimination ability was visualized and quantified through area under the receiver operating characteristic curve (AUROC) and area under the precision-recall curve (AUPRC). The AUROC measures the model’s ability to distinguish between cases and controls across all possible thresholds (0.5 indicates random guessing, 1.0 indicates perfect discrimination). The AUPRC focuses on the trade-off between precision (PPV) and recall (sensitivity), which is particularly useful for imbalanced data sets where CPE-positive cases are rare. To determine if non-linear modeling provided a performance advantage, we benchmarked machine learning algorithms against a standard logistic regression baseline. All models were subjected to identical training and validation procedures. To assess robustness and compare validation strategies, we evaluated the standard random-split models against a separate temporal split. For this temporal validation, new models were trained exclusively on historical data (≤ 2022) and evaluated on a future hold-out set (≥ 2023) to measure performance stability. All computational procedures, including data preprocessing, statistical analysis, and machine learning modeling, were executed in a Python 3.13 environment. The analytical pipeline leveraged *pandas* and *NumPy* for data structuring, while *scikit-learn* provided the framework for model training and performance metrics. Class imbalance was addressed using the *imblearn* library, and Gradient Boosting was implemented via *xgboost*. Visualizations were generated using *matplotlib* and *seaborn* libraries. SHAP (SHapley Additive exPlanations) values were calculated and visualized to determine the contribution of each feature to the prediction probability. Finally, Uniform Manifold Approximation and Projection (UMAP) was employed to visualize the high-dimensional feature space and assess the topological separability of the cases and controls.

### Statistical analysis

Baseline characteristics were summarized using descriptive statistics. For continuous variables exhibiting non-normal distributions, data were expressed as medians with interquartile ranges (IQR). Categorical data were reported as frequencies and proportions. To compare group differences, the χ^2^ test was utilized for categorical comparisons. A two-sided *P* value of <.05 was set as the threshold for statistical significance.

## Results

### Selection of study cohort

**Figure 1.**
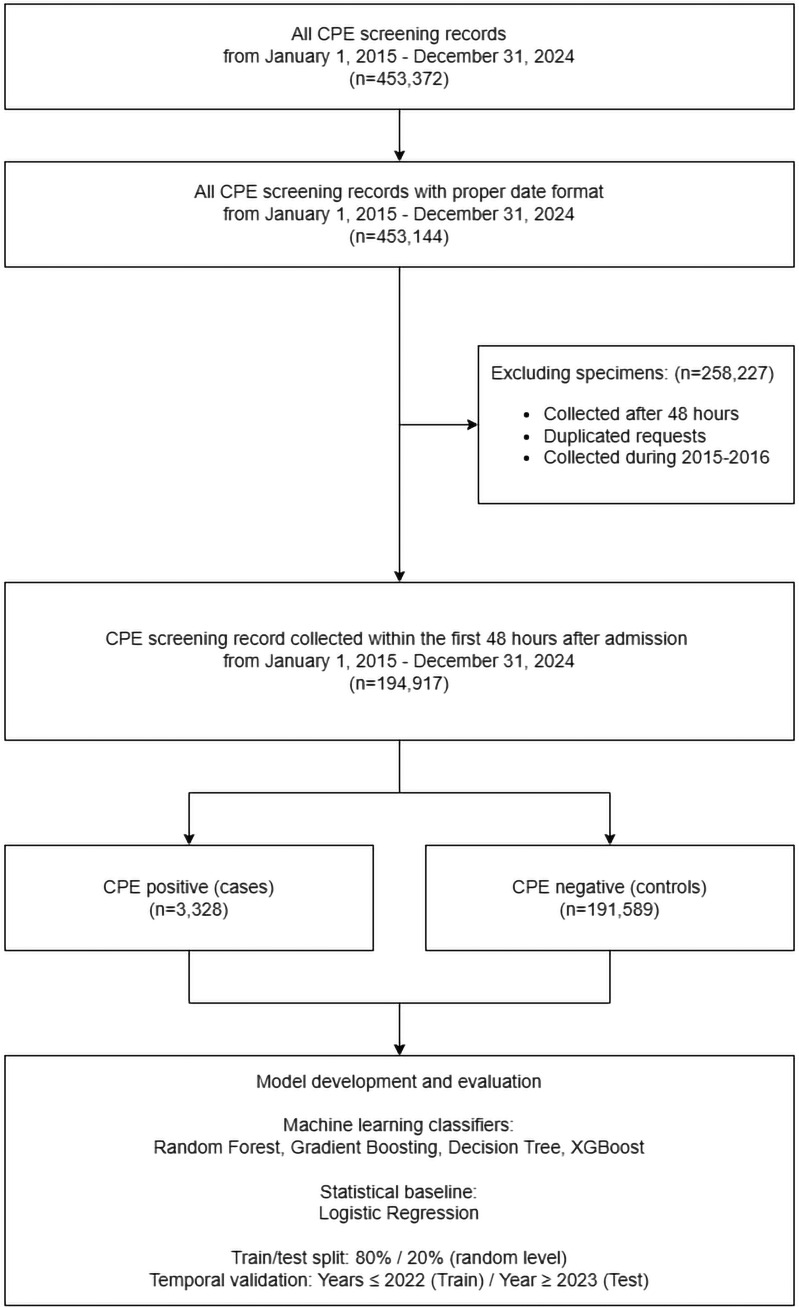
Figure 1 long description. Selection of the study cohort for machine learning. Flow diagram showing inclusion and exclusion of fecal specimens and patient episodes submitted for CPE screening between January 1, 2015 and December 31, 2024, and identification of eligible admission episodes with specimens collected within 48 hours of admission. Data collected from 2015 to 2016 was excluded from the analysis because it served as baseline information for detecting epidemiological parameters related to exposures in the past year. CPE, carbapenemase-producing Enterobacterales.

Between January 1, 2015 and December 31, 2024, a total of 453,372 fecal specimens were submitted for CPE screening in accordance with our infection control protocol. Of these 453,372 screening records, 194,917 (43.0%) fulfilled the inclusion criteria from the fecal specimens collected within 48 hours of admission, which included 3,328 CPE-positive cases (1.7%) and 191,589 CPE-negative controls (98.3%). The remaining 258,455 (57.0%) specimens collected more than 48 hours after admission were excluded. The selection of the study cohort for machine learning is illustrated in Figure 1.

### Baseline characteristics

The baseline characteristics of the study cohort are detailed in Table 1. The admission prevalence of CPE increased significantly over the study period (*P* < .001), increasing from 0.37% (25/6,806) during 2017 to 2.90% (2,055/70,782) by 2023–2024. The median age of CPE-positive cases was slightly lower than that of the control group (65 years [IQR 52–76] vs 68 years [IQR 54–81]; *P* < .001). The proportion of male patients was comparable between the two groups (50.4% in cases vs 50.9% in controls; *P* = .585). Patients with CPE colonization were more likely to have a history of hospitalization in the past year (17.3% vs 12.7%; *P* < .001), while admission from RCHE was less frequent in the case group (7.8% vs 9.8%; *P* < .001).

**Table 1.** Baseline characteristics of patients screened for carbapenemase-producing Enterobacterales (CPE) within 48 hours of admission as input features for machine learning models Table 1 long description.

Characteristic	Overall (N = 194,917)	CPE positive (N = 3,328)	CPE negative (N = 191,589)	*p*-value
Age, years, median [IQR]	68 [54–81]	65 [52–76]	68 [54–81]	<.001
Age group, years, n (%)				
0–17	7,454 (3.8%)	101 (3.0%)	7,353 (3.8%)	.019
18–50	34,769 (17.8%)	663 (19.9%)	34,106 (17.8%)	.002
51–64	41,559 (21.3%)	812 (24.4%)	40,747 (21.3%)	<.001
65–74	41,472 (21.3%)	786 (23.6%)	40,686 (21.2%)	<.001
75 or above	69,663 (35.7%)	966 (29.0%)	68,697 (35.9%)	<.001
Male sex, n (%)	99,164 (50.9%)	1677 (50.4%)	97,487 (50.9%)	.585
Admission from RCHE, n (%)	19,000 (9.7%)	261 (7.8%)	18,739 (9.8%)	<.001
Hospitalization in the past year, n (%)	24,889 (12.8%)	577 (17.3%)	24,312 (12.7%)	<.001
Use of antimicrobials				
Fluoroquinolones use in the past year, n (%)^a^	2,360 (1.2%)	98 (2.9%)	2,262 (1.2%)	<.001
Cephalosporins use in the past year, n (%)^b^	4,554 (2.3%)	184 (5.5%)	4,370 (2.3%)	<.001
Carbapenems use in the past year, n (%)^c^	3,232 (1.7%)	163 (4.9%)	3,069 (1.6%)	<.001
Use of PPIs in the past year, n (%)^d^	14,687 (7.5%)	414 (12.4%)	14,273 (7.4%)	<.001
Known history of CPE colonization in the past year, n (%)	1,113 (.6%)	249 (7.5%)	864 (.5%)	<.001
Comorbidities				
Diabetes mellitus, n (%)	5,769 (3.0%)	74 (2.2%)	5,695 (3.0%)	.013
Hypertension, n (%)	12,882 (6.6%)	189 (5.7%)	12,693 (6.6%)	.032
Renal failure, n (%)	4,891 (2.5%)	105 (3.2%)	4,786 (2.5%)	.019
Malignant tumor, n (%)	16,817 (8.6%)	301 (9.0%)	16,516 (8.6%)	.405
Specialty at admission, n (%)				
Emergency medicine^e^	3,610 (1.9%)	58 (1.7%)	3,552 (1.9%)	.684
General medicine	102,412 (52.5%)	1,442 (43.3%)	100,970 (52.7%)	<.001
Respiratory medicine	1,303 (.7%)	29 (.9%)	1,274 (.7%)	.180
Cardiology	4,949 (2.5%)	80 (2.4%)	4,869 (2.5%)	.657
Nephrology	1,297 (.7%)	45 (1.4%)	1,252 (.7%)	<.001
Hematology^f^	1708 (.9%)	53 (1.6%)	1655 (.9%)	<.001
Pediatrics	2,671 (1.4%)	28 (.8%)	2,643 (1.4%)	.010
Obstetrics and gynecology	2,219 (1.1%)	36 (1.1%)	2,183 (1.1%)	.819
General surgery	35,737 (18.3%)	854 (25.7%)	34,883 (18.2%)	<.001
Orthopedics and traumatology	24,402 (12.5%)	396 (11.9%)	24,006 (12.5%)	.287
Cardiothoracic surgery	5,894 (3.0%)	132 (4.0%)	5,762 (3.0%)	.002
Neurosurgery	3,790 (1.9%)	53 (1.6%)	3,737 (2.0%)	.156
Clinical oncology^g^	1,499 (.8%)	36 (1.1%)	1,463 (.8%)	.047
Liver transplantation	2,374 (1.2%)	54 (1.6%)	2,320 (1.2%)	.039
Blood and marrow transplantation	670 (.3%)	26 (.8%)	644 (.3%)	<.001
Intensive care unit	3,239 (1.7%)	40 (1.2%)	3,199 (1.7%)	.043
Calendar year band, n (% prevalence)				<.001
2017^h^	6,806	25 (.37%)	6,781	
2018–2020	62,600	515 (.82%)	62,085	
2021–2022	54,729	733 (1.34%)	53,996	
2023–2024	70,782	2,055 (2.90%)	68,727	

**Note**. The variables listed in this table constitute the complete set of input features used for the training of the machine learning models and the logistic regression baseline. IQR, interquartile range; PPIs, proton pump inhibitors.

a Fluoroquinolones, including ciprofloxacin, levofloxacin, and moxifloxacin.

b Cephalosporins, including cefazolin, cefuroxime, cefaclor, ceftriaxone, cefotaxime, ceftazidime, cefepime, ceftolozane-tazobactam, ceftazidime-avibactam, and cefiderocol.

c Carbapenems, including meropenem and imipenem-cilastatin.

d Proton pump inhibitors, including omeprazole, esomeprazole, lansoprazole, pantoprazole, and rabeprazole.

e An observation ward is available for the Accident and Emergency Department to provide acute care and to determine if transfer to other clinical specialties is required.

f The Hematology specialty cares for patients with hematological malignancies, excluding blood and marrow transplantation.

g The Clinical Oncology specialty cares for patients with malignancies requiring radiation therapy and chemotherapy, excluding hematological malignancies.

h Data collected from 2015 to 2016 was excluded from the analysis because it served as baseline information for detecting epidemiological parameters related to exposures in the past year.

Prior exposure to antimicrobial agents was notably higher among CPE-positive cases. Specifically, the use of carbapenems (4.9% vs 1.6%; *P* < .001), cephalosporins (5.5% vs 2.3%; *P* < .001), and fluoroquinolones (2.9% vs 1.2%; *P* < .001) in the past year was more prevalent in the case group compared with controls. Similarly, the use of PPIs in the past year was significantly higher in CPE-positive cases (12.4% vs 7.4%; *P* < .001). Notably, a known history of CPE colonization in the past year was a significant risk factor, observed in 7.5% of cases compared with only .5% of controls (*P* < .001).

Regarding comorbidities, renal failure was slightly more frequent in the CPE-positive group (3.2% vs 2.5%; *P* = .019), whereas malignant tumor rates were similar between groups (9.0% vs 8.6%; *P* = .405). In terms of admission specialty, general medicine accounted for the largest proportion of total cases (43.3%), followed by general surgery (25.7%).

### Machine learning model performance

**Figure 2.**
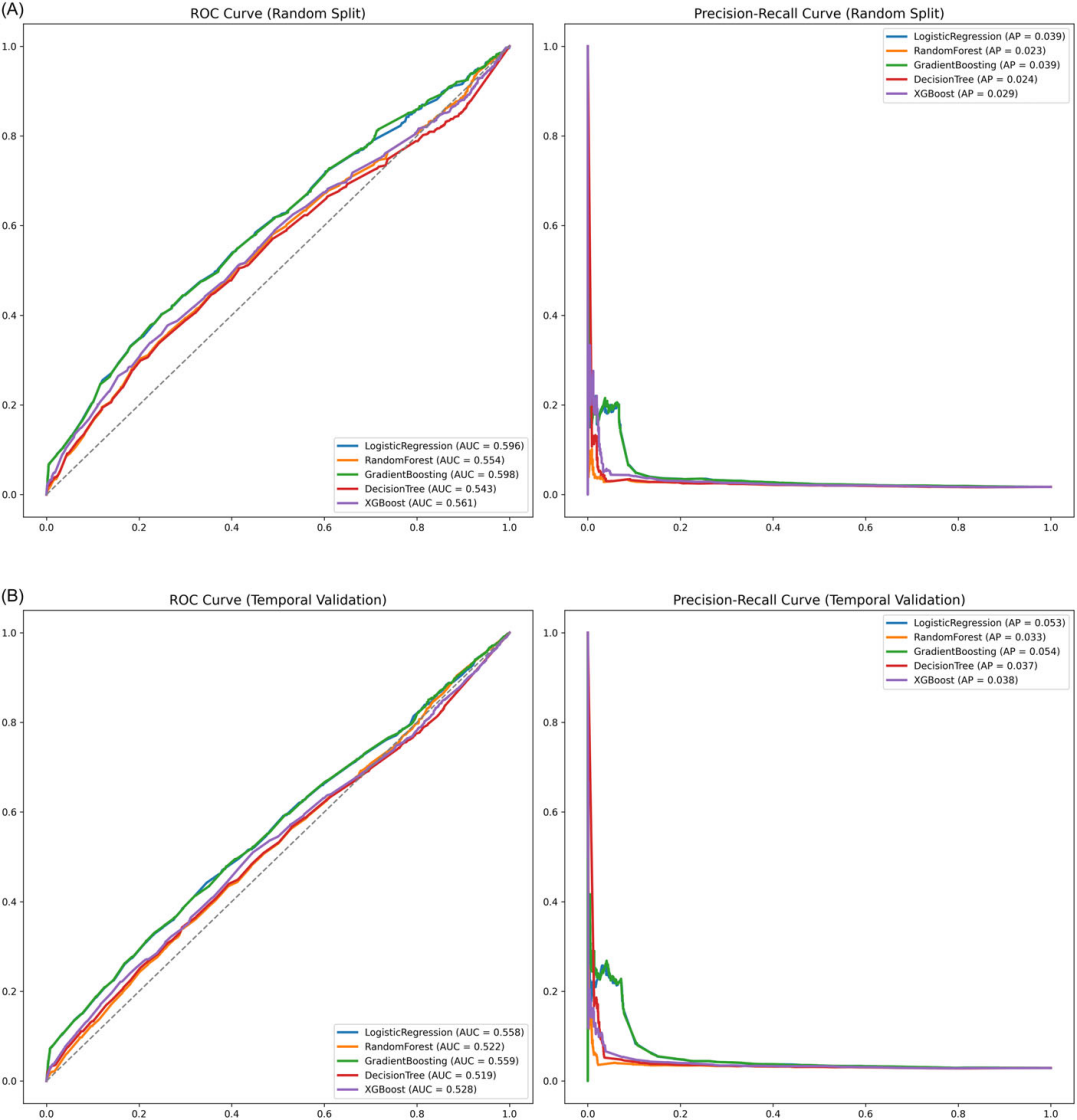
Figure 2 long description. Model performance in predicting CPE colonization within 48 hours of admission. Receiver operating characteristic (ROC) curves for the machine learning classifiers (Gradient Boosting, XGBoost, Random Forest, Decision Tree) and the logistic regression baseline. (A) random split validation: performance on a randomly selected 20% hold-out test set. (B) Temporal validation: performance when training on early study period (2017–2022) and testing on subsequent period (2023–2024). CPE, carbapenemase-producing Enterobacterales.

Table 2 summarizes the performance of the four machine learning models compared with the logistic regression baseline. On the standard 20% random hold-out test set, Gradient Boosting achieved the highest discrimination among the machine learning classifiers (AUROC 0.598), performing comparably to the logistic regression baseline (AUROC 0.596) (Figure 2(A)). This indicates that the increased complexity of the machine learning algorithms did not yield a predictive advantage over standard statistical methods in this cohort.

**Table 2.** Performance of machine learning models compared with the statistical baseline Table 2 long description.

Model Type	Model	Sensitivity	Specificity	PPV	NPV	AUROC	AUPRC
Machine learning models	Gradient Boosting^a^	54.4%	59.1%	2.3%	98.7%	.598	.039
	XGBoost	51.7%	57.2%	2.1%	98.6%	.561	.029
	Random Forest	51.7%	57.0%	2.0%	98.5%	.554	.023
	Decision Tree	50.2%	58.5%	2.1%	98.5%	.543	.024
Baseline	Logistic regression	54.2%	59.3%	2.3%	98.7%	.596	.039

**Note.** PPV, positive predictive value; NPV, negative predictive value; AUROC, area under the receiver operating characteristic curve; AUPRC, area under the precision-recall curve.

a The Gradient Boosting classifier demonstrated the highest discrimination (AUROC) among the machine learning models, performing comparably to the logistic regression baseline.

To assess robustness, temporal validation was performed using the non-overlapping ≥ 2023 hold-out data set. Performance attenuated across all methods; the AUROC for Gradient Boosting decreased to 0.559, mirroring the logistic regression baseline drop to 0.558 (Figure 2(B)). This performance drop indicates that the risk profile of the patient population or the colonization dynamics may have evolved over time.

### Model interpretability

**Figure 3.**
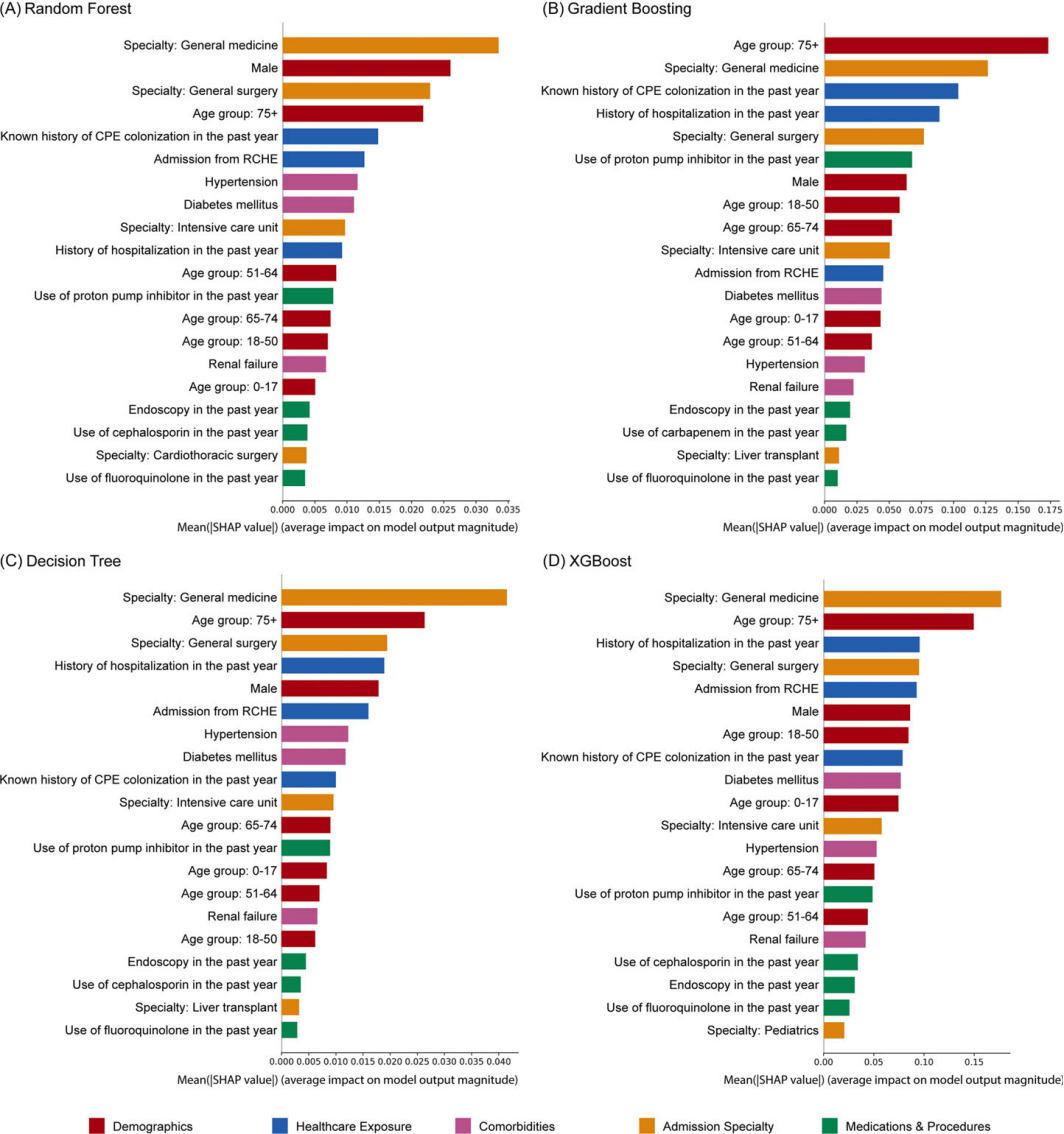
Figure 3 long description. SHAP bar plots illustrating global feature importance for the prediction of CPE colonization. The bars represent the mean absolute SHAP values, indicating the average magnitude of impact each feature has on the model’s output. Only the top predictors of CPE colonization within 48 hours of admission in each model are shown. Features are color-coded by clinical category (Demographics, healthcare exposure, comorbidities, admission specialty, and medications and procedures). CPE, carbapenemase-producing Enterobacterales; RCHE, residential care homes for the elderly; SHAP, SHapley Additive exPlanations.

SHAP bar plots were generated to interpret the global feature importance for each model, with top predictors color-coded by clinical category (Figure 3). Admission to general medicine, age 75 years or older, admission to general surgery, known history of CPE colonization in the past year, history of hospitalization in the past year, and male sex were consistently included in all models as top predictors. Cephalosporin use in the past year was included in the top predictors for the Random Forest, Decision Tree, and XGBoost models, but not in the Gradient Boosting model. Conversely, carbapenem use in the past year was included as a top predictor in the Gradient Boosting model but not in the others. Overall, demographic factors, healthcare exposures, and admission specialty consistently drove the models’ overall predictive power.

**Figure 4.**
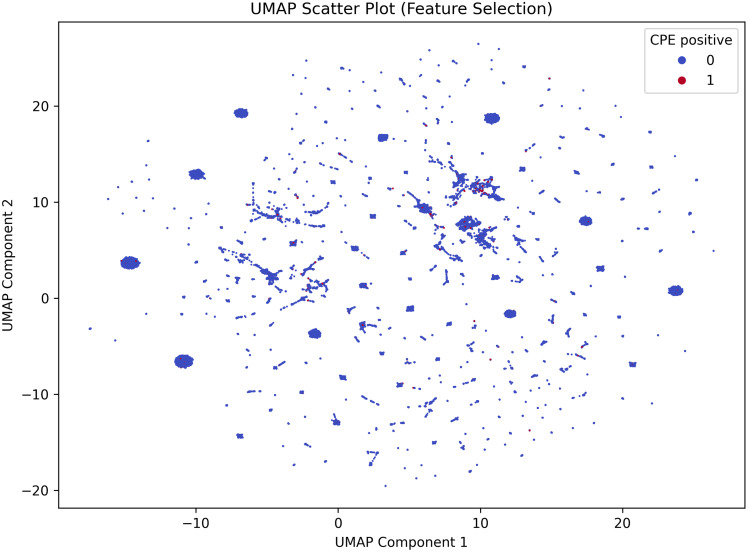
Figure 4 long description. Uniform Manifold Approximation and Projection (UMAP) visualization of the study cohort. Each dot represents a unique patient admission case projected into a two-dimensional space based on the input clinical and demographic features. Red dots represent CPE-positive cases, and blue dots represent CPE-negative controls. Interpretation: the plot reveals a high degree of topological overlap, with CPE-positive cases diffusely distributed throughout the CPE-negative controls rather than forming distinct clusters. This lack of separability visually corroborates the low AUROC values, indicating that the available admission variables are insufficient to distinctively characterize the risk profile of CPE colonization.

To further investigate the challenge in predictive performance, we visualized the data set using UMAP (Figure 4). The projection revealed a high degree of topological overlap between the CPE-positive and CPE-negative populations. Visually, this means that cases did not form a separate group but were mixed indiscriminately with controls. This lack of distinct clustering suggests that the currently available demographic and clinical history variables are insufficient to differentiate the risk profile of CPE colonization.

## Discussion

In this large, single-center cohort, models built from routinely available admission-time demographic and clinical history variables showed limited discriminative ability for predicting CPE colonization (AUROC 0.543–0.598), indicating that these variables alone are insufficient for accurate individual level prediction, despite known epidemiologic associations. This finding aligns with recent analyses of CPE acquisition modeling conducted in an Irish hospital.^18^ In contrast, attempts to develop and validate machine learning models for predicting patients colonized or infected with MDROs upon intensive care unit admission have produced inconsistent results, with patient samples ranging from 800 to 3,500 in those studies.^19–21^ This variability demonstrates the challenges in reliably predicting CPE colonization across different clinical settings.

These findings highlight the complexities associated with utilizing machine learning in infection control. Inconsistencies may arise from factors such as variability in patient populations, the range of input data selected, and the specific algorithms employed. This was supported by our UMAP visualization, which showed substantial overlap between cases and controls. As an unsupervised projection, UMAP is illustrative rather than definitive, but the observed overlap is consistent with the modest AUROC values and suggests limited separability using the available admission-time variables. We evaluated four commonly used tree-based classifiers (Decision Tree, Random Forest, Gradient Boosting, and XGBoost) that are well suited to structured clinical data and compared them against a logistic regression baseline.^22–24^ All models performed comparably, suggesting that the primary limitation was that the available clinical variables lacked sufficient predictive power, rather than the choice of algorithm. However, predictive performance remained low across models. This suggests that although traditional epidemiological factors may be statistically associated with CPE, they provide limited individual level discrimination in a tertiary-care admission population where many exposures are common. Together with the low prevalence of CPE colonization within 48 hours of admission, this challenges the feasibility of a reliable predictive score based solely on standard routinely captured variables. The consistently low PPV is also expected in this context because even moderately specific models will generate many false positives when prevalence is 1.7%. Therefore, any practical use would be more plausibly for risk stratification, relying on the high NPV to rule out low-risk patients, rather than replacing microbiological screening for confirmation.

Given these intricacies, the application of machine learning for predicting CPE colonization upon admission provides a novel perspective in infection control. Traditional epidemiological investigations into the risk factors for CPE colonization have relied on case-control studies employing bivariate and multivariate analyses.^25–30^ These studies identified factors such as prior healthcare exposure, residency in long-term care facilities, previous exposure to antimicrobials or PPIs, and the presence or recent use of invasive devices and procedures as significant associations with CPE acquisition. Prior literature has suggested that establishing a predictive score based on these epidemiological factors could assist in identifying patients at-risk of harboring CPE upon hospital admission in endemic areas.^31^ However, our results temper this expectation, demonstrating that without additional markers, these standard factors are insufficient for precise individual level prediction.

A major limitation in applying machine learning to this domain is the quality and granularity of available data. Although epidemiological information can be retrieved through case record reviews in clinical studies, it is not always represented in computer systems or as big data suitable for machine learning applications. In our study, the primary parameters available in our data set included past admissions, residence in RCHE, and prior use of specific antimicrobial agents and PPIs. Although these factors are relevant, they do not encompass the full spectrum of epidemiologically significant variables. Crucially, our data set lacked information regarding international travel or prior exposure to endemic regions where CPE is prevalent,^32^ as well as exposure to wet markets, which has been significantly associated with CPE acquisition locally.^15^ Such exposures can significantly increase the risk of CPE colonization upon admission; however, our analytical model lacked critical external data. Future research should focus on refining these models by incorporating a more comprehensive set of epidemiological parameters and exploring advanced methodologies to enhance predictive performance.

This study has several limitations. First, as a single-center, retrospective analysis without time-matching, findings may be influenced by temporal shifts in hospital policies and community transmission, limiting generalizability. Second, despite applying SMOTE, the low admission prevalence of CPE (1.7%) inherently constrains predictive performance and results in a low PPV. Third, while a known history of CPE colonization in the past year was correctly identified as one of the top predictors, its use as a standard screening criterion implies that models relying on basic demographics are insufficient for detecting unknown carriers. Fourth, we evaluated standard tree-based models on tabular data; more complex architectures are unlikely to improve results without richer input features like travel history or ward level colonization pressure. Finally, the evolution of diagnostic methods from molecular platforms to lateral flow assays over the study period may have introduced label heterogeneity.

We attempted an initiative to integrate artificial intelligence (AI) in our clinical services, specifically targeting infectious disease management and infection control consultations. The goal was to alleviate the increasing workload of our clinical microbiologists and ICNs, who are essential in monitoring and managing infections in healthcare settings.^33,34^ Despite our efforts, we encountered significant challenges with the currently implemented AI platforms, which did not yield reliable and actionable solutions at this time. The limitations of these systems highlighted the complexities involved in using AI for clinical decision making. Similarly, our machine learning models fell short in providing accurate predictions for CPE, considered a surrogate for MDROs, within 48 hours of admission. This inadequacy emphasized the continued necessity for microbiology laboratory testing to effectively identify patients who are either colonized or infected by CPE at this stage. Consequently, the reliance on traditional diagnostic methods remains vital in ensuring prompt and appropriate patient management.

## Conclusion

This study highlights the challenges associated with applying machine learning for predicting CPE colonization within 48 hours of admission, while also pointing out the limitations of the current models. The low AUROC values indicate that these models may be ineffective in accurately predicting CPE colonization at the individual patient level, likely due to the rarity of such events and the overlap in risk profiles.

## Data Availability

The data sets generated for this study will be made available in anonymized form from the corresponding author upon reasonable request.

## Figure 1 Long description

The flowchart begins with all CPE screening records from January 1, 2015, to December 31, 2024, totaling 453,372 records. It then filters these records to include only those with a proper date format, resulting in 453,144 records. The next step involves excluding specimens collected after 48 hours, duplicated requests, and those collected during 2015-2016, which removes 258,227 specimens. This leaves 194,917 CPE screening records collected within the first 48 hours after admission. These records are then divided into CPE positive cases (3,328) and CPE negative controls (191,589). The final step involves model development and evaluation using machine learning classifiers such as Random Forest, Gradient Boosting, Decision Tree, and XGBoost, with logistic regression as the statistical baseline. The train/test split is 80 percent for training and 20 percent for testing at a random level, with temporal validation for years 2022 and beyond for training and year 2023 and beyond for testing.

Navigate back to Figure 1.

## Figure 2 Long description

The image contains four graphs: two ROC curves and two precision-recall curves. The ROC curves and precision-recall curves are presented for different machine learning classifiers, including Gradient Boosting, XGBoost, Random Forest, Decision Tree, and a logistic regression baseline. The first set of graphs (A) shows performance on a randomly selected 20 percent hold-out test set, while the second set (B) shows performance when training on an early study period from 2017 to 2022 and testing on a subsequent period from 2023 to 2024. Each graph includes multiple lines representing different classifiers, with their respective performance metrics such as AUC and AP values indicated in the legends. The ROC curves plot the true positive rate against the false positive rate, while the precision-recall curves plot precision against recall. The graphs illustrate the comparative effectiveness of the classifiers in predicting CPE colonization.

Navigate back to Figure 2.

## Figure 3 Long description

The image contains four bar graphs labeled Random Forest, Gradient Boosting, Decision Tree, and XGBoost, each illustrating the top predictors of CPE colonization within 48 hours of admission. The bars represent the mean absolute SHAP values, indicating the average magnitude of impact each feature has on the model's output. Features are color-coded by clinical category: Demographics in red, Healthcare Exposure in blue, Comorbidities in purple, Admission Specialty in orange, and Medications and Procedures in green. Each graph lists features such as Specialty: General medicine, Age group: 75+, and Known history of CPE colonization in the past year, among others. The graphs show variations in feature importance across different models, highlighting the most significant predictors for each. All values are approximated.

Navigate back to Figure 3.

## Figure 4 Long description

A scatter plot visualizes patient admission cases using Uniform Manifold Approximation and Projection (UMAP). Each dot represents a unique patient admission case projected into a two-dimensional space based on clinical and demographic features. Red dots indicate CPE-positive cases, while blue dots indicate CPE-negative controls. The plot shows a high degree of topological overlap, with CPE-positive cases diffusely distributed among CPE-negative controls, forming no distinct clusters. This distribution suggests that the available admission variables are insufficient to distinctly characterize the risk profile of CPE colonization, aligning with low AUROC values. The x-axis represents UMAP Component 1, and the y-axis represents UMAP Component 2. All values are approximated.

Navigate back to Figure 4.

## Table 1 Long description

The table presents baseline characteristics of patients screened for carbapenemase-producing Enterobacterales (CPE) within 48 hours of admission, comparing CPE-positive and CPE-negative groups. It includes data on age, age group, sex, admission from residential care home for the elderly (RCHE), hospitalization in the past year, use of antimicrobials, known history of CPE colonization, comorbidities, specialty at admission, and calendar year band. The table has 35 rows and 7 columns, with headers including Characteristic, Overall, CPE positive, CPE negative, and p-value. Notable trends include a significant increase in CPE prevalence from 0.37% in 2017 to 2.90% in 2023-2024, a slightly lower median age for CPE-positive cases, and differences in hospitalization history and antimicrobial use between the groups.

Navigate back to Table 1.

## Table 2 Long description

The table presents a comparison of the performance metrics for four machine learning models against a logistic regression baseline. The models evaluated are Gradient Boosting, XGBoost, Random Forest, and Decision Tree. The metrics include sensitivity, specificity, positive predictive value (PPV), negative predictive value (NPV), area under the receiver operating characteristic curve (AUROC), and area under the precision-recall curve (AUPRC). Gradient Boosting achieved the highest AUROC of 0.598, closely followed by the logistic regression baseline with an AUROC of 0.596. The sensitivity values range from 50.2% to 54.4%, while specificity values range from 57.0% to 59.3%. PPV values are consistently low, around 2.0% to 2.3%, and NPV values are high, around 98.5% to 98.7%. This indicates that the machine learning models did not significantly outperform the logistic regression baseline in this cohort.

Navigate back to Table 2.
